# Multicenter Evaluation of BD Max Enteric Parasite Real-Time PCR Assay for Detection of Giardia duodenalis, Cryptosporidium hominis, Cryptosporidium parvum, and Entamoeba histolytica

**DOI:** 10.1128/JCM.00765-16

**Published:** 2016-10-24

**Authors:** S. Madison-Antenucci, R. F. Relich, L. Doyle, N. Espina, D. Fuller, T. Karchmer, A. Lainesse, J. E. Mortensen, P. Pancholi, W. Veros, S. M. Harrington

**Affiliations:** a Parasitology Laboratory, Wadsworth Center, NYSDOH, Albany, New York, USA; b Indiana University School of Medicine, Indianapolis, Indiana, USA; c Cleveland Clinic, Cleveland, Ohio, USA; d Becton Dickinson and Company, Sparks, Maryland, USA; e Becton, Dickinson and Company, Quebec, Quebec, Canada; f Cincinnati Children's Hospital, Cincinnati, Ohio, USA; g The Ohio State University Wexner Medical Center, Columbus, Ohio, USA; University of Tennessee

## Abstract

Common causes of chronic diarrhea among travelers worldwide include protozoan parasites. The majority of parasitic infections are caused by Giardia duodenalis, Entamoeba histolytica, Cryptosporidium parvum, and Cryptosporidium hominis. Similarly, these species cause the majority of parasitic diarrhea acquired in the United States. Detection of parasites by gold standard microscopic methods is time-consuming and requires considerable expertise; enzyme immunoassays and direct fluorescent-antibody (DFA) stains have lowered hands-on time for testing, but improvements in sensitivity and technical time may be possible with a PCR assay. We performed a clinical evaluation of a multiplex PCR panel, the enteric parasite panel (EPP), for the detection of these common parasites using the BD Max instrument, which performs automated extraction and amplification. A total of 2,495 compliant specimens were enrolled, including 2,104 (84%) specimens collected prospectively and 391 (16%) specimens collected retrospectively. Approximately equal numbers were received in 10% formalin (1,273 specimens) and unpreserved (1,222 specimens). The results from the EPP were compared to those from alternate PCR and bidirectional sequencing (APCR), as well as DFA (G. duodenalis and C. parvum or C. hominis) or trichrome stain (E. histolytica). The sensitivity and specificity for prospective and retrospective specimens combined were 98.2% and 99.5% for G. duodenalis, 95.5% and 99.6 for C. parvum or C. hominis, and 100% and 100% for E. histolytica, respectively. The performance of the FDA-approved BD Max EPP compared well to the reference methods and may be an appropriate substitute for microscopic examination or immunoassays.

## INTRODUCTION

The latest surveillance data tracking the global burden of diseases indicate that among communicable conditions, lower respiratory tract infections and diarrheal disease contribute the highest number of disability-adjusted life years (1). Infections with Giardia duodenalis (also referred to as G. lamblia and G. intestinalis), Cryptosporidium hominis, Cryptosporidium parvum, and Entamoeba histolytica are common parasitic causes of diarrheal disease and are found in both low-income and high-income countries. These parasites have been identified as significant causes of foodborne illness. A World Health Organization study recently reported that the enteric protozoa contributed to 67.2 million illnesses or 492,000 disability-adjusted life years (2, 3). In 2012, there were 7,956 cases of cryptosporidiosis and 15,178 cases of giardiasis reported to the CDC (4). There may be a perception that infections with intestinal parasites are extremely rare in the United States; however, it is interesting to note that the number of cases of diarrheal illness caused by Cryptosporidium spp. and Giardia duodenalis is greater than reported episodes of intestinal illness caused by Shiga-toxin producing Escherichia coli (6,463 episodes) and episodes caused by Shigella species (15,283 episodes) (4).

While amebiasis is not tracked nationally, some states do have surveillance programs, which provide data on the number of reported cases of diarrheal disease associated with intestinal amoebae. The number of amebiasis cases reported are not necessarily exclusively due to E. histolytica or *Entamoeba histolytica/E. dispar* but do provide an estimate of amebiasis. Both New York and California annually record slightly higher numbers for infections with intestinal amoebae than with Cryptosporidium species. Therefore, the total number of cases nationally may be greater than 8,000 per year. Taken together, the annual number of reported cases of infections with intestinal parasites is more than 32,000, and the actual number of infections is likely to be much higher.

Despite a significant burden of disease due to infections with intestinal protozoa, few commercially available modern methods of detection have been developed. Traditional means of identifying parasites depend on microscopic examination of stained slides. Stool samples are typically concentrated, slides are generated and stained using one or more methods best suited to the specific organism, and then they are viewed manually under the microscope. The methods require expertise that is difficult to fulfill, as clinical parasitologists and training are limited in the United States. Microscopy can also be time-consuming, particularly for less-experienced technologists. Even in experienced hands, microscopic examination has limited sensitivity, as only a small portion of a sample is viewed.

While immunochromatographic or lateral flow and enzyme immunoassays (EIAs) are available for the detection of intestinal protozoa, there are drawbacks to these quick and easy methods. For example, low sensitivity and high false-positive rates have been reported when testing for Cryptosporidium spp. using rapid tests (5, 6). As a result, the case definition for cryptosporidiosis was changed in 2010 to consider positive results by rapid tests as presumptive rather than confirmed. Direct fluorescent-antibody (DFA) stains for Cryptosporidium and Giardia offer greater sensitivity over permanent stains (7). Yet, DFA still requires significant hands-on time, particularly compared to automated molecular methods. Rapid tests for Entamoeba spp. can only be used on unfixed stool samples, and many of the assays cannot distinguish pathogenic E. histolytica from E. dispar, which is typically considered nonpathogenic. However, a recent report suggests that E. dispar is capable of causing lesions (8).

Here, we evaluate an automated multiplex real-time PCR assay for the detection of commonly encountered and clinically significant G. duodenalis, C. hominis, C. parvum, and E. histolytica in formalin-fixed and unpreserved stool specimens. The performance of the BD Max enteric parasite panel (EPP) was compared to that of DFA, trichrome staining, and conventional PCR combined with bidirectional sequencing. Although G. duodenalis is currently the accepted species name with regard to human infections, users will note that the BD Max EPP package insert refers to G. lamblia.

## MATERIALS AND METHODS

### Clinical samples.

A total of 2,495 deidentified remnant stool specimens from adult and pediatric patients suspected of having a parasitic gastrointestinal infection were evaluated in this study. Enrolled specimens were assigned a unique study number with no relationship to any identifiable portion of the patient name, date of birth, medical record number, or other identifying numbers. The only information collected was the patient's age group (≥2, 3 to 12, 13 to 21, or >21 years). All specimens were enrolled in accordance with institutional review board (IRB) protocols at the collection site. Specimens excluded were those submitted on swabs, those in preservatives other than 10% formalin, those from patients undergoing antiparasitic therapy, those from individuals for whom parasitological investigations were not ordered, and retrospective specimens without original test results. Specimens were collected from clinical centers (*n* = 5) in the United States, collection centers in Mexico, Uganda, and the United States (*n* = 3), and several specimen banks. A total of 2,104 (84%) specimens were collected prospectively between July 2013 and April 2014, of which 1,126 (54%) specimens were preserved and 978 (46%) specimens were unpreserved. An additional 391 (16%) specimens collected in 2012 and 2013, comprising 147 (38%) preserved specimens and 244 (62%) unpreserved specimens, were included in the retrospective analysis.

Unpreserved prospective specimens were stored at 25 ± 2°C, 2 to 8°C, or −20°C, depending on whether they were tested with the BD Max EPP within 48 h, 120 h, or 30 days from collection, respectively. Preserved prospective specimens were stored at 25 ± 2°C or between 2 and 8°C if they were to be tested within 120 h or 30 days of collection, respectively. Unpreserved retrospective specimens were stored frozen (−20°C or −80°C) until thawed and were then tested within 48 h if stored at 25 ± 2°C or 120 h if stored between 2 and 8°C after thawing. Preserved retrospective specimens were stored at 2 to 8°C prior to testing.

### Spiked samples.

E. histolytica was poorly represented in clinical specimens. Therefore, 100 E. histolytica trophozoite-spiked stools and 100 control negative stools were included in the study. E. histolytica strain HM-1:IMSS trophozoites were axenically cultured, as previously described (9). Cells were enumerated using a hemocytometer, and various concentrations of E. histolytica trophozoites representing 100, 50, 10, 4, and 2 times the limit of detection (LOD) were added to formalin-preserved and unpreserved E. histolytica-negative stools. For unpreserved samples, the LOD was 16.8 organisms/ml in sample buffer tubes (SBTs) or 2,519 organisms/ml in the original unpreserved stool (9). The LOD for formalin-fixed samples was 15.5 organisms/ml in sample buffer or 9,300 organisms/ml in the original preserved stool specimen, reflecting the 1:4 dilution in 10% formalin.

### Reference methods.

All prospective clinical specimens were tested by a direct fluorescent-antibody (DFA) test, according to the manufacturer's instructions, using the MeriFluor Cryptosporidium/Giardia kit (Meridian, Cincinnati, OH). This method detects Cryptosporidium spp. and the Giardia duodenalis cyst stage in smears of concentrated preserved specimens. Microscopic examination of trichrome-stained polyvinyl alcohol-fixed smears were utilized for the detection of Entamoeba histolytica. Microscopic examination was conducted either by the clinical centers participating in the study or by a reference laboratory, Microbiology Specialists, Inc. (Houston, TX), if a center was not able to perform microscopy for one or more parasites.

Alternate PCR that targeted distinct nucleic acid sequences from those of the BD Max EPP assay, followed by bidirectional sequencing of amplicons (APCR), was performed on both prospective and retrospective samples. For APCR, 178-bp, 175-bp, and 338-bp regions of the small-subunit rRNA genes of Cryptosporidium spp., Giardia spp., and Entamoeba spp., respectively, were amplified based on previously described methods (10–12). All nucleic acid extraction and amplification reactions were performed by BD Diagnostics (Baltimore, MD). Briefly, extraction was performed using the Roche MagNA Pure LC total nucleic acid isolation kit (large volume) on the Roche MagNA Pure LC 2.0 system. One milliliter of sample, comprising 400 μl of remnant sample from the BD Max EPP SBT combined with 600 μl of BD Max EPP sample buffer, was loaded into each well of the MagNA Pure cartridge. Extraction was then performed as per the manufacturer's instructions. Ten microliters of the 100-μl final eluate was used for subsequent PCR amplification. Amplicons were referred to ILS Genomics, LLC (Research Triangle Park, NC) for bidirectional sequencing and final result disposition. Samples were called positive by sequencing if the trace quality score was ≥25, E scores were ≤10^−30^, top BLAST hits included C. parvum or C. hominis, G. duodenalis, or E. histolytica, and samples had 2× bidirectional coverage with a minimum of 100 contiguous overlapping base pairs. The average (minimum to maximum) overlap for positive specimens was 171 bp (163 to 182 bp) for Cryptosporidium, 165 bp (125 to 177 bp) for Giardia, and 331 bp (329 to 334 bp) for Entamoeba, respectively. Overlap was similar between samples and positive controls. For retrospective specimens, the historical reference result was confirmed with APCR and bidirectional sequencing to rule out specimens that had been contaminated or misidentified, or where the target organism had degraded during storage.

### BD Max EPP analysis.

Testing of all specimens was performed according to the manufacturer's instructions. Briefly, using calibrated disposable inoculating loops, 10-μl aliquots of vortexed stool sample were transferred into SBTs. The SBTs contain 1.5 ml of a proprietary sample diluent that is formulated to minimize PCR inhibition associated with stool specimens. To ensure extraction of nucleic acids from encysted protozoans, all specimens in SBTs were heated using the BD Max prewarming heater. Samples are heated gradually to 114°C and then cooled prior to transfer to the BD Max sample racks. The entire prewarming step takes about 52 min. Following the prewarming step, manufacturer-provided unitized reagent strips, which contain all of the reagents required for nucleic acid extraction and amplification, and a microfluidics cartridge were loaded onto the BD Max instrument. Five hundred microliters of the diluted sample is transferred to the lysis tube of the unitized reagent strip. Sample transfer and subsequent steps are performed automatically by the BD Max instrument. Primers included in the BD Max EPP assay detect G. duodenalis, Cryptosporidium (C. hominis and C. parvum only), and E. histolytica. Cryptosporidium is reported as positive or negative, but species is not identified. Genetic targets and sequences are proprietary. Amplified products are detected with fluorophore-labeled TaqMan probes. The instrument monitors fluorescent signals at each PCR cycle, and internal software provides automatic interpretation of results (13). The user does not adjust the threshold for a positive result or interpret cycle threshold (*C_T_*) values. The total run time for the EPP, including sample processing, the prewarming incubation, PCR, and result reporting is 4.5 h for a batch of 24 samples.

### Controls and unresolved results.

All extraction and amplification runs included one negative and one positive external control. The positive external control contained genomic DNA for Giardia intestinalis (Lambl) Alexeieff ATCC 30888D, C. parvum Tyzzer ATCC PRA-67D, and E. histolytica ATCC 30459D. An internal control was included with each test specimen. If results were not reportable due to a lack of amplification for the internal control or either of the external controls, the test was repeated using the initially inoculated SBT. If the controls failed on the repeat amplification, a second SBT was prepared, and up to 2 additional extraction and amplification reactions could be performed.

### Environmental monitoring.

Prior to study initiation and weekly thereafter, each testing site performed environmental monitoring using the BD Max EPP. Ten work area locations, including instrumentation, were sampled. If a positive result was obtained, the area or equipment was decontaminated. Negative results from testing of repeated sampling were required prior to reinitiating clinical specimen testing.

### Data analysis and discrepancy testing.

Data were first analyzed by comparing results from the BD Max EPP to reference microscopic and APCR methods separately. Additionally, the results of the BD Max EPP were assessed using a composite reference method. For G. duodenalis, the definition of a positive composite reference result is based on an “or” algorithm. A positive result by either DFA or APCR is a positive reference result. Since DFA and trichrome stains detect species of Cryptosporidium and Entamoeba, respectively, in addition to those targeted by the BD Max EPP, interpretation of reference results is more complex for these genera. To ensure the validity of the DFA results for Cryptosporidium spp., all samples yielding a positive result by either DFA or the BD Max EPP were repeated by DFA. In addition, 12% of all specimens that were negative by both DFA and APCR were repeated. If the repeat DFA confirmed the original result, no additional testing was performed. If the repeat did not confirm the original result, a 3rd DFA test was performed. The concordant DFA test result was recorded as the final result. If DFA was positive but APCR identified a species other than C. parvum or C. hominis, the composite reference result was negative. However, if the DFA was positive and the APCR was negative, the reference result was positive. If the DFA was negative and APCR identified C. parvum or C. hominis, the reference result was positive. For E. histolytica, the definition of a positive composite reference sample was based on an “and” algorithm. Since nonpathogenic species are difficult to distinguish from E. histolytica microscopically, samples that were positive by trichrome staining but negative by APCR were considered negative.

Several methods were used to investigate discrepant results between the BD Max EPP and reference methods, and these methods included repeat testing of the BD Max EPP, DFA, and APCR, as well as enzyme immunoassay testing by Giardia/Cryptosporidium Chek and E. Histolytica II (TechLab, Blacksburg, VA).

Sensitivity and specificity were calculated with 95% confidence intervals using the reference methods as the gold standard. Prevalence rates for each target were calculated as the number of prospective specimens that tested positive by the composite reference method divided by the total number of compliant trial specimens.

## RESULTS

A total of 2,495 compliant specimens were evaluated; 2,104 specimens were collected prospectively, and 391 were retrospective samples. Age information was unknown for 10.6% of the samples and was not available for retrospective samples. Among those collected prospectively, the majority of specimens were from adults over the age of 21. Children under 12 represented 18.3% of samples; teens and young adults comprise another 10.8% of the specimens. Although chronological data were collected, the sample size was not large enough to perform statistical analyses. However, no trends in sensitivity or specificity were observed due to age.

### Prevalence.

Five U.S. laboratories that perform clinical testing were selected as test sites to enroll and test specimens using their normal methods as well as the BD Max EPP. One study site was excluded from prevalence calculations, as it is a state public health laboratory and receives prospective specimens that are presumptively positive for enteric parasites. For the four remaining U.S. sites, prevalences ranged from 0.3% (2/592) to 8.7% (2/23) for G. duodenalis and from 0% (0/23) to 8.4% (36/427) for C. parvum and C. hominis. Both the highest prevalence for Giardia and the 0% prevalence for Cryptosporidium were based on a relatively small sample size of 23 specimens enrolled at one site. Prevalence at all other U.S. sites was based on enrollment of between 292 and 592 specimens. Excluding the state public health lab, overall prevalence calculated based on prospective specimens was 2.4% for G. duodenalis (42/1,786 specimens) and 2.7% for C. parvum and C. hominis (47/1,770 specimens). No E. histolytica was detected among the prospective specimens (1,404 specimens).

In addition to the U.S. sites, samples were collected in Mexico and Uganda. By country of specimen origin, prevalence was as follows: 2.1% for G. duodenalis (33/1,602) and 4.6% for C. parvum and C. hominis for the United States (73/1,587); 0% for G. duodenalis, based on 169 specimens, and 0% for C. parvum and C. hominis for Mexico, based on 164 specimens; and 11.2% (13/116) for G. duodenalis and 31% (35/113) for C. parvum and C. hominis for Uganda.

### Prospective specimens.

The results of samples collected prospectively are given in Table 1. Some samples did not meet study criteria for reference methods for all organisms. For example, a specimen that was preserved in 10% formalin may not have been accompanied by a corresponding sample preserved in polyvinyl alcohol (PVA) to allow trichrome staining for Entamoeba. Therefore, the number of samples evaluated for each target organism varies. The results from the BD Max EPP are compared to those of microscopy, APCR, and an algorithm, referred to as the composite method, wherein the results of both the microscopy and the APCR were considered.

**TABLE 1:** Results of prospective specimens

Organism and specimen type	No. of specimens					% (95% CI)^*a*^	
	True positive	False positive	True negative	False negative	Total	Sensitivity	Specificity
G. duodenalis							
vs DFA	29	12	1,651	2	1,694	93.5 (79.3–98.2)	99.3 (98.7–99.6)
vs APCR	32	9	1,653	0	1,694	100 (89.3–100)	99.5 (99.0–99.7)
Composite	38	3	1,651	2	1,694	95.0 (83.5–98.6)	99.8 (99.5–99.9)
C. parvum or C. hominis							
vs DFA	76	17	1,573	7	1,673	91.6 (83.6–95.9)	98.9 (98.3–99.3)
vs APCR	87	6	1,579	1	1,673	98.9 (93.8–99.8)	99.6 (99.2–99.8)
Composite	88	5	1,576	4	1,673	95.7 (89.3–98.3)	99.7 (99.3–99.9)
E. histolytica							
vs trichrome staining	0	0	1,404	2	1,404	0 (0–65.8)	100 (99.7–100)
vs APCR	0	0	1,404	0	1,404	ND	100 (99.7–100)
Composite	0	0	1,404	0	1,404	ND	100 (99.7–100)

a 95% CI, 95% confidence interval; ND, not determined.

For G. duodenalis, if either microscopy or APCR was positive, the composite method was considered positive. BD Max EPP testing gave 12 false-positive results compared to DFA and nine compared to APCR. Among these, nine of the DFA-negative specimens were positive by APCR, and six of the nine APCR-negative specimens were positive by DFA. Thus, in a comparison of the composite results, only three specimens were negative by both DFA and APCR. There were only two false negatives; both were negative by BD Max EPP and positive by DFA. Repeat testing by DFA was positive for only one, and APCR was negative for both. Repeat testing by BD Max EPP was negative in multiple replicates. Specificity for G. duodenalis with the BD Max EPP was >99% for all methods, and sensitivity ranged from 93.5 to 100%.

For Cryptosporidium, the composite method included repeat testing on all DFA-positive specimens and on all BD Max EPP false-positive specimens. To be considered positive by DFA, the specimen was required to test positive two out of three times. The composite result was considered negative if APCR identified a Cryptosporidium species other than C. parvum or C. hominis, as the BD Max EPP is designed to detect only these two species. A positive DFA or a positive APCR result was included as a positive composite reference result. Comparing BD Max EPP to DFA, there were 17 false-positive specimens. Twelve of the 17 specimens were also positive by APCR, and one APCR-negative specimen was positive by DFA. Thus, five specimens were considered false positive by the BD Max EPP compared to the composite algorithm. One of these was identified by APCR as Cryptosporidium meleagridis and another as a species other than C. parvum or C. hominis. DFA testing was positive for both of these specimens, as the antibodies are not species specific. For the remaining three specimens, only the BD Max EPP was positive.

Comparing the BD Max EPP to DFA, there were seven false negatives for Cryptosporidium. Replicate repeat BD Max EPP for each of the false negatives confirmed the initial results. Two specimens were identified as Cryptosporidium *canis* by APCR, and a third was identified as a Cryptosporidium species other than C. hominis or C. parvum. The results for these specimens are consistent with the positive DFA and negative BD Max EPP results and are considered true negatives. One specimen was positive for C. parvum by APCR on repeat testing, and three specimens were positive only by DFA. Thus, four specimens were considered false negative. Interestingly, two samples that were positive for Cryptosporidium only by DFA were also determined to be positive for G. duodenalis by both DFA and BD Max EPP on repeat testing. The sensitivity of the BD Max EPP for C. parvum and C. hominis ranged from 91.6% compared to microscopy to 98.9% compared to APCR. Specificity with the assay was ≥98.9% for all methods.

For Entamoeba, there were two false-negative specimens, and these were detected by trichrome staining only. Neither was positive for E. histolytica by the BD Max EPP, nor was an Entamoeba species detected by APCR.

### Retrospective specimens.

Due to the low prevalence of the targets in prospective specimens, retrospective specimens were also utilized. Characterization of retrospective specimens may include assays other than those included in the composite algorithm. The results of all retrospective samples (positive and negative) were confirmed by APCR prior to enrollment. As shown in Table 2, testing for G. duodenalis resulted in seven false-positive results. For five of the specimens, no further testing could be performed. The remaining two specimens were positive by EIA and also positive in repeat testing by the BD Max EPP assay in several replicates. In one case, 6 of 6 replicates were positive, and in the other, five of 11 replicates were positive. Only one false negative was encountered, and no further testing of that specimen was possible. The sensitivity for G. duodenalis was 99.2%, and the specificity was 96.6%.

**TABLE 2:** Results of retrospective specimens

Organism	No. of specimens					% (95% CI)^*a*^	
	True positive	False positive	True negative	False negative	Total	Sensitivity	Specificity
G. duodenalis	127	7	200	1	335	99.2 (95.7–99.9)	96.6 (93.2–98.4)
C. parvum or C. hominis	83	3	259	4	349	95.4 (88.8–98.2)	98.9 (96.7–99.6)
E. histolytica	11	0	245	0	256	100 (74.1–100)	100 (98.5–100)

a 95% CI, 95% confidence interval.

For C. hominis and C. parvum, there were three false-positive results, and additional sample to resolve discrepant results was available for two specimens. One specimen was tested by EIA and found to be negative. Two specimens were tested repeatedly by the BD Max EPP and were negative in six out of six replicates. The results suggest that the initial positive result may have been incorrect. There were four false-negative results. Residual sample was available for repeat testing on all four specimens. The BD Max EPP yielded positive results for only two of the specimens. In one case, 5 of 12 replicates were positive, and in the other, only 2 of 12 replicates were positive. The results suggest that these specimens contained Cryptosporidium at levels close to the limit of detection for the assay. Sensitivity was 95.4%, and specificity was 98.9%.

Fifty-three retrospective specimens that were positive for Entamoeba were enrolled in the study (Table 3). APCR and bidirectional sequencing confirmed the presence of Entamoeba histolytica in only 11 unpreserved specimens and none of the formalin-fixed specimens. Ten of the 11 specimens were from Mali, and one was from Canada; all 11 were positive by the BD Max EPP.

**TABLE 3:** Species identified by sequencing of specimens positive for Entamoeba spp. by alternate PCR

Entamoeba species	No. of specimens
E. dispar	22
E. coli	19
E. hartmanni	9
E. gingivalis	2
E. polecki	1
E. bovis	1
E. muris	1

### Results for spiked specimens.

Two hundred specimens (100 formalin-fixed and 100 unpreserved) were generated as described above using trophozoites of E. histolytica. Fifty of each specimen type were spiked with E. histolytica at various concentrations above the limit of detection (LOD). Half of the spiked specimens were 2-fold above the LOD, and the remaining specimens were spiked at various concentrations up to 100-fold above the LOD. As negative reference samples, 50 of each specimen type were unspiked. All specimens yielded expected results, giving 100% sensitivity and 100% specificity. The cycle threshold values obtained for spiked samples were similar to those observed in the retrospective segment. The median *C_T_* for study specimens was 25.2. The minimum and maximum *C_T_* values were 19.8 and 33.5, respectively. The median *C_T_* value for spiked specimens in 10% formalin was 24.7, with a range of 19.2 to 27.8. For unpreserved spiked samples, the median *C_T_* value was 23.5, and the range was 18.4 to 28.7. Thus, the spiked specimens were a good representation of the number of parasites observed in the patient specimens.

### Species detected by bidirectional sequencing from alternate PCR.

The BD Max EPP was designed to detect specific species, including C. parvum, C. hominis, and E. histolytica. Yet, samples may contain other species that can contribute to positive DFA and trichrome results. The inclusion of alternate PCR and bidirectional sequencing as a reference method provided additional information on species specificity. Tables 3 and 4 list species of Entamoeba and Cryptosporidium detected by APCR. Importantly, all specimens yielding Entamoeba species other than those targeted by the BD Max EPP tested negative, as expected. Fifty-five samples had nonpathogenic Entamoeba species, with the largest numbers being E. dispar (*n* = 22) and E. coli (*n* = 19). In contrast, 95% of Cryptosporidium specimens contained oocysts of the species that cause the majority of human infections (14). In the current sample set, C. hominis was detected in 46% of the specimens and C. parvum in 49%. Other Cryptosporidium species are also known to cause infection in humans (15) and were identified by APCR in a small percentage of the cases, C. canis (2.7%), C. meleagridis (1.6%), and C. felis (0.5%) (6, 16). One specimen that could only be identified as a species other than C. hominis or C. parvum initially tested positive with the BD Max EPP. Repeat testing of that specimen gave positive results in five out of six replicates. A second sample that tested positive by the BD Max EPP was determined to be C. meleagridis by APCR.

**TABLE 4:** Species identified by sequencing of specimens positive for Cryptosporidium sp. by alternate PCR

Cryptosporidium species	No. (%) of specimens
C. hominis	85 (46.2)
C. parvum	90 (48.6)
C. canis	5 (2.7)
C. meleagridis	3 (1.6)
C. felis	1 (0.5)

### Unpreserved and formalin-fixed specimens.

Samples enrolled in the study were either unpreserved or preserved in 10% formalin and stored as described above. Table 5 shows the results of BD Max EPP compared to the composite reference results for each specimen type. Slightly better results were obtained with formalin-fixed samples when testing for G. duodenalis and with unpreserved samples when testing for C. parvum and C. hominis. Formalin fixation may slightly reduce the lysis of oocysts prior to amplification. However, there were no statistically significant differences between formalin-fixed and unpreserved specimens.

**TABLE 5:** Comparison of BD Max EPP assay to composite algorithm for unpreserved and formalin-fixed samples

Organism and specimen type	No. of samples					% (95% CI)^*a*^	
	True positive	False positive	True negative	False negative	Total	Sensitivity	Specificity
G. lamblia							
10% formalin fixed	76	3	1,067	1	1,147	98.7 (93.0–99.8)	99.7 (99.2–99.9)
Unpreserved	89	7	784	2	882	97.8 (92.3–99.4)	99.1 (98.2–99.6)
C. parvum or C. hominis							
10% formalin fixed	95	2	1,029	7	1,133	93.1 (86.5–96.6)	99.8 (99.3–99.9)
Unpreserved	76	6	806	1	889	98.7 (93.0–99.8)	99.3 (98.4–99.7)
E. histolytica							
10% formalin fixed	0	0	881	0	881	ND	100 (99.6–100)
Unpreserved	11	0	768	0	779	100 (74.1–100)	100 (99.5–100)

a 95% CI, 95% confidence interval; ND, not determined.

### Performance of controls.

Unresolved results due to failure of the internal control could be caused by inhibitory substances in the stool specimens or reagent failure. For unpreserved specimens, the unresolved rate was 4.7%, while for formalin-fixed specimens, only 1.5% of the results were unresolved. Overall, unresolved results were 2.9% before and 0.5% after all repeat testing. Specimens yielding repeatedly unresolved results were excluded from the performance calculations.

### Overall performance.

To detect intestinal parasites, we have compared conventional methods, such as acid-fast staining, trichrome staining, and direct fluorescent-antibody immunomicroscopy, with an automated molecular detection assay, the BD Max EPP. Discrepant results were resolved by repeat testing and alternate PCR and sequencing. By taking into account the results from microscopy as well as APCR, a composite reference method or algorithm was developed for each of the parasites. Using the composite reference method and comparing the performance of the BD Max EPP across all sample types yields a sensitivity of 98.2% and specificity of 99.5% for G. duodenalis. For C. parvum and C. hominis, the sensitivity was 95.5%, and the specificity was 99.6%. For E. histolytica, sensitivity and specificity were 100%.

## DISCUSSION

As demonstrated in other studies, molecular tests can increase the detection of pathogens while lowering both hands-on time and time to results (17–20). The lack of skilled clinical parasitologists and need to perform multiple tests to adequately test for the relevant parasites adds to the challenge. For microscopic detection, DFA of preserved concentrated stool specimens has the highest sensitivity and specificity for the detection of Giardia and Cryptosporidium, while trichrome-stained smears are the standard for the detection of Entamoeba. The size and shape of Giardia observed by DFA are distinctive and would yield few false positives. In contrast, Cryptosporidium spp. that do not cause disease in humans are not necessarily distinguished from the human pathogens by DFA, and Entamoeba histolytica is differentiated from nonpathogenic E. dispar only in the very rare cases where ingested red blood cells are observed. Although neither is common in the United States, both Entamoeba moshkovskii and Entamoeba bangladeshi are morphologically indistinguishable from E. histolytica and have been associated with disease (21, 22). Additionally, other nonpathogenic intestinal amoebae can be difficult to accurately identify. Minor morphological details are used to distinguish trophozoites of E. histolytica and Entamoeba coli; Entamoeba hartmanni has been referred to as the “little E. histolytica.” As a result, the microscopic detection of these parasites is not only time-consuming but requires extensive experience for accurate identification.

Here, we have compared these conventional methods with the enteric parasite panel on the BD Max system, which performs automated DNA extraction and amplification. The BD Max EPP assay is a multiplex real-time PCR assay designed to detect the G. duodenalis, E. histolytica, C. parvum, and C. hominis, which are common and clinically significant intestinal parasites that cause diarrhea both in the United States and worldwide. To ensure specificity and provide comparison to another molecular test, the BD Max EPP was also compared to an alternate PCR assay that included bidirectional sequencing of amplicons. To maximize the rigor of the evaluation, the BD Max EPP was compared to a composite reference result, as detailed previously.

Specimens tested prospectively are presumed to yield results most representative of those produced by operations in a diagnostic laboratory. The BD Max EPP improved the detection of G. duodenalis over the conventional DFA assay. Not surprisingly, there was better agreement between results from APCR and the BD Max EPP. Nine of the DFA-negative samples were positive by APCR, and six of the samples negative by APCR were positive by DFA, leaving only three false positives by the BD Max EPP when both DFA and APCR were taken into account. Specificity for the BD Max EPP was 99.8%. Based on prospective specimens, the positive predictive value (PPV) was 92.7%, and the negative predictive value (NPV) was 99.9%. Samples with inconsistent reference test results may have contained a low level of Giardia that was detected by the BD Max EPP but not by APCR or DFA. At the time of this study, FDA-approved molecular tests were not available for additional arbitration of discrepant results.

A recent study (23) compared the BD Max EPP to microscopy and to a lab-developed PCR assay for a small set of clinical samples. The sensitivity for G. duodenalis was reported as 66.7% (8/12) compared to PCR and 100% (18/18) compared to microscopy. In that study, the threshold values for the BD Max EPP had been set based on a limited number of samples, some of which were spiked. Evaluation of the amplification curves generated with authentic clinical samples revealed that a fluorescent signal clearly above background was present in some specimens originally called negative by the software. The fluorescent cutoff utilized in the BD Max EPP was subsequently optimized by BD to improve sensitivity while maintaining high specificity. Our study utilized proprietary software incorporating the optimized cutoff, which is the threshold in the current FDA-approved assay.

For Cryptosporidium, the results from the BD Max EPP correlated better with APCR than DFA (Table 1). Consistent with the greater sensitivity of molecular methods, 12 specimens that were negative by DFA were positive by APCR. One of the specimens that was positive by BD Max EPP was identified as C. meleagridis by APCR. A second could not be identified to the species level, but both C. hominis and C. parvum were ruled out. Thus, it is possible that the BD Max assay could give a positive result for other species of Cryptosporidium. Overall, only 8 Cryptosporidium specimens detected by reference methods were species that less frequently cause infections in humans, C. canis (5 specimens), C. meleagridis (3 specimens), and C. felis (1 specimens) (Table 4). This is in agreement with the often-reported statistic that 90 to 95% of cryptosporidiosis is caused by C. hominis and C. parvum. Contact with farm animals and domestic dogs and cats has occasionally been shown to be a risk factor for the acquisition of zoonotic species (24–26). While the detection of such species might be considered an advantage of DFA, this advantage is offset by its lower sensitivity. For prospective specimens, the BD Max EPP gave a PPV of 94.6%, an NPV of 99.7%, and identified 12 positive specimens that were missed by DFA.

Unfortunately, only 2 of 2,104 prospective specimens tested positive for Entamoeba, and these were only detected by microscopy. It is likely that these were not Entamoeba histolytica. However, as the APCR was negative, the species could not be identified. With the goal of increasing the number of positive specimens, 165 prospective specimens were obtained from Mexico and 106 specimens from Uganda (data not shown). The prevalences of Giardia and Cryptosporidium were highest in specimens from Uganda (11.2% and 31%, respectively). However, none of the Ugandan specimens were positive for Entamoeba, and specimens obtained from Mexico were negative for all three parasites. We were surprised by the lack of positive results but have no explanation for this finding. Fortunately, 11 retrospective specimens were positive for E. histolytica. The BD Max EPP detected all 11 samples; there were no false-negative or false-positive results for this parasite. In contrast, 55 nonpathogenic amoebae were detected by APCR, with 28 from retrospective samples and 27 from samples collected prospectively (Table 3). This highlights the problem posed by microscopic detection of intestinal amoebae, since the number of samples with E. dispar was double that of E. histolytica. If results for these specimens were reported based on microscopy or other tests that do not differentiate the species, only one-third would actually be due to infection with the pathogenic parasite. Additionally, there were 19 specimens that were positive for E. coli, and many of these could be mistaken for E. histolytica if the result were based solely on morphology.

Another advantage of the BD Max EPP is that it can be performed with specimens submitted in either 10% formalin or unpreserved. The sensitivity and specificity for Giardia and Cryptosporidium were similar for both sample matrices (Table 5). Since there were no samples positive for Entamoeba in formalin-fixed samples, limited conclusions regarding this matrix may be drawn from the spiked specimen data. These results demonstrated successful extraction of DNA from parasites and detection of nucleic acids in the presence of formalin. However, only trophozoites were utilized, and the effects of prolonged transport or storage were not evaluated.

A time study analysis was not included in this evaluation. However, as discussed above, microscopic methods are notoriously time-consuming and require considerable technical expertise. Our data support the use of the BD Max EPP as a rapid multiplex molecular test that may be implemented in a clinical laboratory to improve sensitivity compared to conventional microscopic methods. Since the BD Max assay targets pathogenic species of Cryptosporidium and Entamoeba, the BD Max EPP also has the advantage of improving specificity for these targets.
